# Supplementary material for: SyNDI: synchronous network data integration framework
Source: BMC Bioinformatics. 2018 Nov 6;19:403. doi: 10.1186/s12859-018-2426-5 (PMC6219086; doi:10.1186/s12859-018-2426-5)
Supplement: Supplementary file 5 — Files of the example presented in Additional file 4. This additional file is a zip package that contains all files used in Additional file 4. Detailed descriptions of each of these files are presented in a README file included in this zip package. (GZ 30411 kb) [file 12859_2018_2426_MOESM5_ESM.gz › SyNDITools/Meme2Fimo/meme_4.9.0_4.tar.gz!/meme_4.9.0/doc/meme-install.html]

Installing the MEME System


# MEME Suite

The MEME Suite web application requires the use of JavaScript
  
Javascript doesn't seem to be available on your browser.

# Installing MEME

# (version 4.6.0 or later)

### Table of Contents:

- Quick Install
- Introduction
- Prerequisite software
- Getting the source
- Getting and installing the patches
- System requirements
- MEME Suite directories
- Installing from a tarball
- Installing from an SVN repository
- Parallel MEME
- Customizing configuration
- Installation Examples
- Setting up MEME Web Application Under Tomcat
- Uninstalling
- Using MEME Suite
- Running regression tests
- Installing sequence databases
- Installing GOMO databases
- Installing motif databases
- Known issues
- Reporting problems

---

## Quick Install (for Linux, OS-X and Cygwin systems)

1. Type the following commands and then follow the instructions
   printed by the configure command.
     
   $ tar zxf meme\_VERSION.tar.gz
     
   $ cd meme\_VERSION
     
   $ ./configure --prefix=$HOME/meme --with-url=http://meme.nbcr.net/meme
     
   $ make
     
   $ make test
     
   $ make install
2. Edit your shell configuration file to add
     
   $HOME/meme/bin
   to your shell's path.

**Notes:**

- This installs only the command-line versions of programs. Refer
  to Installing from a tarball
  if you wish to install your own MEME Suite Web Application as well.
- This will overwrite any previously existing
  $HOME/meme directory
  you may have. You can set the directory to something different in
  the configure command using the
  --prefix option.
- The HTML output of programs will contain buttons that send your
  data to
  http://meme.nbcr.net
  for additional processing when
  clicked. Refer to the Installing from a tarball
  if you do not want this feature.

---

## Introduction

The MEME Suite consists of several command line programs and an optional
web site. The web site is implemented using static HTML pages,
CGI scripts, and SOAP services.
The SOAP services are provided through
Tomcat,
and Opal.
Building and installing the command line programs is very straight-forward.
Installing the web site requires more detailed configuration information.

## Prerequisite software

Most of the software mentioned here is standard on many UNIX systems.
To build MEME Suite you will need:

- Perl (v.5.10.1 or higher) is needed to run some scripts.
  See http://www.perl.org  for download
  and installation instructions.
- The following perl modules: (there are others but they are typically already
  available as part of the Perl installation.)

- HTML::PullParser
- HTML::Template

- Python (v2.6 up to v2.7.x) is needed to run some scripts (including DREME).
  See http://www.python.org for download
  and installation instructions. Pypy (http://pypy.org/)
  is also compatible with MEME and may offer a speed boost.
- Bourne compatible shell
- C-shell compatible shell
- Make utility
- C compiler
- Gzip/gunzip utilities

If you are installing the web site you will also need:

- A web server supporting CGI. Apache
  is a common choice.
- Version 5.0.30 or later of the Apache Tomcat
  servlet container and web server.
  Version 5.5.25 is recommended because it supports CGI, and allows Tomcat to
  host the entire MEME Suite web application. MEME Suite has not been tested
  with version 6.
- Version 2 of Opal SOAP
  wrapper servlet.
- The following perl modules: (there are others but they are typically already
  available as part of the Perl installation.)

- HTTP
- HTML::PullParser
- HTML::Template
- LWP
- SOAP::Lite
- XML::Simple

Optional software:

- Version 2.6.0 or later of the libxml2 library.
  If this library is not available
  the MEME Suite will build a copy for its own use, but having this library already installed will
  greatly speed up the build process.
- Version 1.1 or later of the libxslt library.
  If this library is not available
  the MEME Suite will build a copy for its own use, but having this library already installed will
  greatly speed up the build process.
- The convert program from the ImageMagick package is needed to convert logograms
  from EPS to PNG format .
- - A Linux cluster running **Rocks** 
    (see http:www.rocksclusters.org )
    or similar cluster environment. **Rocks** provides all the required and
    optional software.
  - If you want to run parallel version of MEME you will need an MPI
    implementation and a batch scheduler. For MPI, one of the following:
    - LAM from http://www.lam-mpi.org
    - MPICH from http://www-unix.mcs.anl.gov/mpi/mpich
    - OpenMPI from http://www.open-mpi.org
    For batch scheduler, one of the following:
    - SGE from http://gridengine.sunsource.net
    - PBS from http://www.openpbs.org

[ Top ]

---

## Getting the source

The current version of MEME is 4.5.0 and is available from http://meme.nbcr.net/downloads/.

**Note:**
MEME has undergone many changes in version 4.0.0. It is optimized and
configured for running on Linux servers. It hasn't been fully tested on other platforms,
and your feedback is greatly appreciated. If you'd like to wait until all the kinks to be
worked out before trying the current release, please use the previous version
(use version 3.5.7) from http://meme.nbcr.net/downloads/old\_versions.
Installation instructions for version 3.5.7 are different and are
specified in the README file included in the release distribution meme\_3.5.7.tar.gz.

The software is distributed as a source tarball
meme\_VERSION.tar.gz. For each file there is a corresponding file
with the MD5 checksum: meme\_VERSION.md5sum. When downloading the
software, please make sure you download the corresponding checksum file and verify
that the checksum information is correct. Depending on a platform and
available software, the verification command can be different. Here there are 3
possible ways to do verification:

1. Using md5sum command, usually available on Linux.
     
   $ md5sum meme\_VERSION.tar.gz
2. Using md5 command, usually available on Solaris.
     
   $ md5 meme\_VERSION.tar.gz
3. Using openssl command, usually available on any UNIX.
     
   $ openssl dgst -md5 meme\_VERSION.tar.gz

The output of the command (numerical part) should be exactly the same as one provided in the
checksum file. Here is the checksum information for available MEME versions:

| File | md5sum |
| --- | --- |
| meme\_4.3.0.tar.gz | please see meme\_4.3.0.md5sum |
| meme\_4.2.0.tar.gz | please see meme\_4.2.0.md5sum |
| meme\_4.1.0.tar.gz | please see meme\_4.1.0.md5sum |
| meme\_4.0.0.tar.gz | please see meme\_4.0.0.md5sum |
| meme\_3.5.7.tar.gz | please see meme\_3.5.7.md5sum |
| meme\_3.5.4.tar.gz | please see meme\_3.5.4.md5sum |
| meme\_3.5.1.tar.gz | please see meme\_3.5.1.md5sum |
| meme\_3.5.0.tar.gz | cb552052c82c40ce3111c95f1e9697eb |
| meme\_3.0.14.tar.gz | f18bacdce60c11bff4cde650da296228 |

[ Top ]

---

## Getting and installing the patches

The distribution may have patches associated with it.
They are available from http://meme.nbcr.net/downloads/.
Patch files are located in a directory named
VERSION.patches,
for example, meme\_3.5.4.patches.
Patch file have names like:
VERSION.patch\_SERIAL\_NO,
for example, meme\_3.5.4.patch\_3.

To install a patch, download the patch file from the URL given above.
Then perform the following commands to install it:
  
$ cp PATCH\_FILE VERSION 
  
$ cd VERSION 
  
$ patch -p1 < PATCH\_FILE 
  
$ make install 
  
$ make test

For example, to install the first patch to version meme\_3.5.4, you
would perform the following commands:
  
$ cp meme\_3.5.4.patch\_1 meme\_3.5.4 
  
$ cd meme\_3.5.4 
  
$ patch -p1 < meme\_3.5.4.patch\_1 
  
$ make install 
  
$ make test

You must install all of the patches for a specific version in serial
number order. For example, if you wish to install patch number 3,
you must first have installed patches number 1 and 2 for that version.
This is easy to do. Just download all the patches for your current
version, copy them to your current version's directory, and then
install them in order by repeating the patch,
command above, with each patch file. You only need to run the
install and
make test commands once, after
the last patch command.

[ Top ]

---

## System Requirements

MEME has been used successfully on the following platforms

- v 4.0.0
  - Redhat Enterprise Linux, release 5
  - Fedora Linux 7
  - MacOS X 10.5 (Leopard)
  - Cygwin (DLL 1.5.19-4.) running under Windows
  - v 3.5.7
    - Redhat Enterprise Linux, release 4
    - MacOS X 10.4.4 (Tiger)
    - Cygwin (DLL 1.5.19-4.) running under Windows
    - v 3.5.1
      - Redhat Enterprise Linux, release 4
      - MacOS X 10.4.4 (Tiger)
      - Cygwin (DLL 1.5.19-4.) running under Windows
      - SunOS 5.9
    - v 3.5.0
      - SunOS 5.9
      - Redhat Enterprise Linux, release 4

Disk use:

- Minimal installation including only command line tools: 2.7 MB
- Full web site (not including sequence databases) 23 MB

[ Top ]

---

## MEME Suite Directories

Installation of MEME Suite will create the following directories:

```
                bin/    db/    etc/    lib/    LOGS/    web/
```

The directories contain:

**bin/**
:   MEME Suite executables and scripts

**db/**
:   this directory or DIR specified by --with-db=DIR option is created.
    Initially, the database directory is empty, and is filled with the
    sequence databases after running update\_db command.

**doc/**
:   Supplementary documentation for some of the MEME Suite tools.

**etc/**
:   contains the following files

    - db\_\*.csv - sequence database information listed as comma separated
      values. Used for running update\_db command
    - mast.doc, meme.doc - text files used by a few commands to provide
      usage info
    - prior1.plib, prior30.plib - these files are used by MEME

**lib/**
:   contains common Perl modules

**LOGS/**
:   directory for temporary files created by mast or MEME when jobs are
    submitted via web

**web/**
:   contains static HTML and CGI scripts needed for a web-based access to
    the MEME Suite programs. This directory is only created if the MEME
    Suite web site is installed.

[ Top ]

---

## Installing from a tarball

It is recommended to create a **meme** user prior to installation.
Put the tarball you obtained from the web site in a directory where you
want to build the software. If the software distribution was obtained
via SVN access please see section
Installing from an SVN repository.

Four steps are required to install the MEME Suite from a distribution tarball:

1. Unzip and untar the distribution
2. Configure the installation using the configure command.
3. Build MEME Suite using the make command
4. Install MEME Suite using the make install command

### 1. Unzip and untar the distribution:

Use the commands:

$ tar zxf meme\_VERSION.tar.gz
  
$ cd meme\_VERSION

to expand the tarball and move to the root directory of the distribution.

If there are patches associated with the VERSION please see section
Getting and installing the patches that explains how to
download and install the patches.

### 2. Configuring the installation

The configure script is found
in the root directory of the distribution and used
to set installation parameters. For a list of all available
configuration parameters see section
Customizing configuration below.
Here we will discuss the most common configuration settings.
The general form of the configure command is:

$ ./configure [parameters here]

**Basic configuration parameters**

When invoked without any parameters,
only the command-line versions of the MEME Suite tools will be installed
and none of the web components.
The MEME Suite directories
will be created under $HOME.
The buttons and hyperlinks in the HTML output of the tools
will not work if you use this command (unless you also install
a MEME Suite web server on the same host). This is safe (you
won't be able to send your data over the internet accidentally),
but not very useful. However, if this is what you want the
simplest command is:

$ ./configure

MEME Suite tool output files contain buttons that will send your results
over the Internet when clicked. This is a very useful and powerful feature
of the MEME Suite tools.
If you want the buttons and hyperlinks in the HTML output of the tools
to access the MEME Suite web server at NBCR,
the simplest configuration command is

**$ ./configure --with-url="http://meme.nbcr.net/meme"**

The installation root directory can be specified using
the parameter prefix.
For example,

**$ ./configure --prefix=your-install-path --with-url="http://meme.nbcr.net/meme"**

This will install only the command-line versions of the MEME Suite tools
and none of the web components.
The MEME Suite directories will be
created under the directory specified by the prefix option.
The buttons and hyperlinks in the HTML output of the tools
will link to the MEME Suite web server at
NBCR.
**This is probably the most common use of configure.**

The MEME Suite depends on the libraries
libxml2
and
libxslt.
If these libraries are already installed,
MEME will use try to use the installed versions.
Otherwise, MEME will build its own copies of these libraries.
If for some reason you want to force MEME Suite to build its own
copies of libxml2
and libxslt,
you can do so by using the
enable-build-libxml2
and
enable-build-libxslt
parameters.
For example,

$ ./configure \
  
--prefix=your-install-path \
  
--enable-build-libxml2 \
  
--enable-build-libxslt

This will cause the MEME Suite to build its own copies of
libxml2
and
xslt.

**Configuring installation of the MEME Suite Web Application**

Four parameters are needed to completely configure the MEME Suite web application:

- the URL for the web site hosting the static HTML pages and CGI scripts
- the URL for the web site hosting the MEME Suite SOAP services
- the name of the directory where the static HTML pages and CGI scripts
  should be installed
- the name of the directory containing the Opal build file for configuring
  the MEME Suite SOAP service

In many cases you will be able to take advantage of the default values for these parameters.

The parameter with-url
was introduced in the previous section.
It sets the URL for the web site hosting the static HTML pages and CGI scripts.
If you are installing your own MEME Suite web application you will almost
certainly want to set this parameter.

The parameter enable-web
turns on installation of the MEME Suite web site.
It can also be used to specify the URL for the MEME Suite SOAP services.
The default URL for the MEME Suite SOAP services is
http://ws.nbcr.net/opal2/services.
For example,

$ ./configure \
  
--prefix=your-install-path \
  
--with-url=url-for-meme-on-your-web-server \
  
--enable-web

would install the MEME Suite command line tools and the web application under
your-install-path. The web application would use
the MEME Suite SOAP services at
http://ws.nbcr.net/opal2/services.
to process requests.

An explicit value can also be passed for the
enable-web parameter.
For example, to explicitly specify the value for the MEME Suite SOAP services on the NBCR server:

$ ./configure \
  
--prefix=your-install-path \
  
--with-url=<url-for-meme-on-your-web-server> \
  
--enable-web=http://ws.nbcr.net/opal2/services/

The parameter with-webdir
configures the installation step so that the static HTML pages and CGI scripts
will be copied to a particular directory.
It defaults to <your-install-path>/web.
For example, to force the static HTML and CGI files to be
installed in /var/www/meme
you would use the command:

$ ./configure \

Alternatively you can use a symbolic link to mount the web directory
in the web servers directory tree (see step 4 below).

If you wish to build and install your own MEME Suite SOAP services you must
have successfully installed and configured
Tomcat,
and Opal.

You must set the environment variable CATALINA\_HOME to the path to the
root directory of Tomcat:

|  |  |
| --- | --- |
| $ setenv CATALINA\_HOME tomcat-path | # for csh, tcsh |
| or | |
| $ export CATALINA\_HOME=tomcat-path | # for bash, sh |

The parameter enable-webservice
is used to specify the location of the ANT build file for
Opal.

For example, to install the website and build and provide using your own
MEME Suite SOAP services:

$ ./configure \

### 3. Building

To build simply type while at the top directory:

$ make

You can run tests to make sure the MEME Suite programs are working correctly.
The test will check MEME Suite
output against known valid output:

$ make test

If there are errors, the output files from each test will be kept in
`tests/results/` and can be compared to files in `tests/`.
When tests pass without errors the `results/` with the output files
is removed automatically.

### 4. Installing

To install the software in the directory tree specified by `prefix`
run the command:

$ make install

**Configuring apache web access**

This part requires root access to create a symbolic link from the document root
directory to the web directory in MEME installation. For example, if meme
software is installed in `/home/meme`, and the HTTP
`DocumentRoot` is `/var/www/html`, then create
a link:

The URL for web access is typically `http://your.host.name/meme/`.
Virtual hosts may be configured accordingly.

[ Top ]

---

## Installing from a SVN repository

The process of configuring, building, and installing MEME Suite from an SVN
repository is the same as
installing from a tarball, but with
two extra, initial steps: you must check out the repository, and
you must build the configure script.

Access to the SVN repository is granted at this point only to the
MEME developers. We provide the tarball distribution of the latest version
on the ftp site.

If you have SVN access, you can check out the source code with the command:

$ svn checkout svn+ssh://meme@summit.ucsd.edu/meme/SVNROOT/trunk

Once you've checked out the source code you need to build the
configure script:

$ cd trunk

You can now follow the configuration, building and install steps
from installing from a tarball.

[ Top ]

---

## Parallel MEME

By default, `configure` will establish if your system has the right
software to produce parallel version of MEME. Parallel compilation is disabled if
mpicc is not found or if queue status program `qstat` (part
of the SGE or PGS scheduler) is not found.
Both must be present on the system. Please see section
Prerequisite software  for information where to get
the software. Currently, parallel version of MEME is known to work with:

| MPI distributions | Batch Schedulers |
| --- | --- |
| LAM MPI | SGE |
| MPICH | PBS |
| OpenMPI |  |

- If your mpicc is installed in non-standard place, use
  `--with-mpidir=MPIDIR` option when
  running 'configure' to specify the MPI installation directory.
- If you have a custom version of mpicc (for example, one from
  Sun HPC ClusterTools) that provides wrappers use
  `--with-mpicc=MPICC` option to specify mpicc or its wrapper by full path.
  The configure looks by default only for names 'mpicc' and 'mpcc'.

Once `mpicc` and `qsub` are located by configure,
the parallel version of MEME, called `meme_p` is compiled and installed
automatically via commands `make` and `make install`.

[ Top ]

---

## Customizing configuration

Customization is done via the command line arguments to configure.
To find out a list and the syntax of the configurable arguments, execute:
$ ./configure --help

The general syntax for all the options is one of the following:

- Include a **feature**:
    
  --enable-**feature**
    
  or
    
  --enable-**feature**=yes
- Do not include a **feature**:
    
  --disable-**feature**
    
  or
    
  --enable-**feature**=no
- Set a specific **feature** to a **value**:
    
  --with-**feature**=**value**

The following table provides a list of parameters
available for `configure`:


| Option | Needed for | Default value |
| --- | --- | --- |
| --prefix | Provide the installation location path. | $HOME |
| --enable-build-libxml2 | Forces MEME Suite to build and use libxml2 code included in MEME Suite distribution. | disabled |
| --enable-build-libxslt | Forces MEME Suite to build and use libxslt code included in MEME Suite distribution. | disabled |
| --enable-debug | Produce an executable with debugging symbols. | no debug |
| --enable-opt | Produce an executable with optimization. | compiler and OS dependent. |
| --enable-serial | Produce only serial version of MEME, do not build parallel version. | disabled |
| --enable-web[=http://url.to.meme/webservice] | Build web site applications. Must point it to MEME Suite web services exposed via Opal. Defaults to http://ws.nbcr.net/opal2/services/ | disabled |
| --enable-webservice=/path/to/build.xml | Deploy MEME Suite web services utilizing an existing Opal installation. Must point to the build file for the Opal installation. | disabled |
| --with-contact=EMAIL | Set email of web site administrator. | $USER@$HOSTNAME |
| --with-convert=path-to-convert-binary | Set location of ImageMagick convert application. | As found in PATH |
| --with-db=DBDIR | Set path to databases. | install-path/db/ |
| --with-dot=path-to-dot-binary | Set location of Graphviz dot application. | As found in PATH |
| --with-logs=LOGDIR | Set path to logs. | install-path/LOGS/ |
| --with-maxtime=TIME | Set wall time limit (sec) a web-submitted job started by the web server may use. | 7200 |
| --with-mpicc=MPICC | Set MPICC to use. | found mpicc |
| --with-mpidir=MPIDIR | Set MPI installation path. | path of found mpicc |
| --with-perl=path-to-perl-binary | Set location of Perl interpreter. | As found in PATH |
| --with-python=path-to-python-binary | Set location of Python interpreter. | As found in PATH |
| --with-procs=numproc | Set number of processors to use for parallel jobs. | 2 |
| --with-url=URL | Set url for the MEME Suite web site used by buttons on the output of MEME Suite tools. | http://$HOSTNAME/meme |
| --with-blocks-url=URL | Set url for the BLOCKMAKER web site. | http://blocks.fhcrc.org/blocks-bin/process\_blocks.pl |
| --with-goserver-url=URL | Set url for the AMIGO web site. | http://amigo.geneontology.org/amigo |
| --with-webdir=DIR | Set path to MEME Suite web site directory. | install-path/web/ |

**Note 1**: see  Parallel MEME section for details.

[ Top ]

---

## Installation Examples

1. **Example 1**: installing MEME command line programs without any web
   components in /opt/meme. Buttons in the MEME output will submit to the default
   remote server: NBCR.
     
   $ tar zxf meme\_4.3.0.tar.gz
     
   $ cd meme\_4.3.0
     
   $ ./configure --prefix=/opt/meme
     
   $ make
     
   $ make test
     
   $ make install
   - **Example 2**: installing MEME command line programs without any web
     components in /opt/meme. Buttons in the MEME output will submit to the
     URL: `http://meme.imb.uq.edu.au/`.
       
     $ tar zxf meme\_4.3.0.tar.gz
       
     $ cd meme\_4.3.0
       
     $ ./configure --prefix=/opt/meme --with-url=http://meme.imb.uq.edu.au/
       
     $ make
       
     $ make test
       
     $ make install
   - **Example 3**: installing MEME command line programs and the static
     HTML and CGI scripts, but not the SOAP services.
     The URL for the MEME web site will be `http://host/meme` and
     buttons in the MEME output will submit to that URL.
     This installation will use the MEME SOAP services on
     http://ws.ncbr.net/opal2/services.
       
     $ tar zxf meme\_4.3.0.tar.gz
       
     $ cd meme\_4.3.0
       
     $ ./configure --prefix=/home/meme --enable-web --with-url=http://host/meme
       
     $ make
       
     $ make test
       
     $ make install
   - **Example 4**: installing the full MEME Suite
     including all the command line programs, the static HTML and CGI scripts,
     and the SOAP services. The static HTML, CGI scripts, and SOAP services
     will all be served by an installation of
     Tomcat.
     `http://host/meme`.
       
     $ tar zxf meme\_4.3.0.tar.gz
       
     $ cd meme\_4.3.0
       
     $ export CATALINA\_HOME=/home/meme/tomcat
       
     $ ./configure \
       
      --prefix=/home/meme \
       
      --enable-web=http://host:8080/opal/services
       
      --enable-webservice=/home/meme/opal/build.xml \
       
      --with-url=http://host:8080/meme \
       
      --with-webdir=/home/meme/tomcat/webapps/meme
       
     $ make
       
     $ make test
       
     $ make install
   - **Example 5**: installing the full MEME Suite
     including all the command line programs, the static HTML and CGI scripts,
     and the SOAP services. The static HTML, and CGI scripts will be served by
     an APACHE web server. The SOAP services will all be provided by a separate
     installation of
     Tomcat.
       
     $ tar zxf meme\_4.3.0.tar.gz
       
     $ cd meme\_4.3.0
       
     $ export CATALINA\_HOME=/home/meme/tomcat
       
     $ ./configure \
       
      --prefix=/home/meme \
       
      --enable-web=http://host:8080/opal/services
       
      --enable-webservice=/home/meme/opal/build.xml \
       
      --with-url=http://host/meme \
       
      --with-webdir=/home/meme/Sites/meme
       
     $ make
       
     $ make test
       
     $ make install

[ Top ]

---

## Setting Up MEME Web Application Under Tomcat

Note: This procedure was tested with Tomcat 5.0.30.

1. In $CATALINA\_HOME/webapps, make
a symbolic link to the MEME web installation directory. For example
  
$ cd $CATALINA\_HOME/webapps
  
$ ln -s $MEME\_INSTALL\_DIR/web meme

2. In $CATALINA\_HOME/conf/Catalina/localhost,
create a new file meme.xml with the following
contents.
  
<Context path="/meme" docBase="meme" debug="0" allowLinking="true">
  
</Context>

3. In $CATALINA\_HOME/conf/web.xml, uncomment
the block of code just below "Common Gateway Includes (CGI) processing servlet, which supports...".
Set param-value to 
cgi-bin. In addition, uncomment the block of code right below
"The mapping for the CGI Gateway servlet".

4. Rename $CATALINA\_HOME/server/lib/servlets-cgi.renametojar
 to $CATALINA\_HOME/server/lib/servlets-cgi.jar.

5. Restart Tomcat.

[ Top ]

---

## Uninstalling

You can unistall MEME Suite by giving the command:
  
$ make uninstall  
in the root of the build directory. This will remove all
the files installed for the MEME Suite, but won't remove
any directories that may have been created.

[ Top ]

---

## Using MEME Suite

Set the environment variable PATH to newly installed MEME Suite executables:

|  |  |
| --- | --- |
| $ setenv PATH your-install-path/bin:${PATH} | # for csh, tcsh |
| or | |
| $ export PATH=your-install-path/bin:$PATH | # for bash, sh |

[ Top ]

---

## Running regression tests

There is a suit of tests that can be run to test MEME Suite output
against known output. These tests can be run during the installation
process. A quick "smoke test" can be run using the command:
  
$ make test   
A more complete set of tests can be run from the `tests` subdirectory of the
build directory:
  
$ cd tests
  
$ ../scripts/runtests  
This full set of tests can take a long time to run.
The output files from the tests go into the newly created
directory `tests/results/`. If the tests pass without
errors this directory with its output files will be deleted. If there are
errors the output files are kept and can be compared to files in
tests.

[ Top ]

---

## Installing sequence databases

If you have installed MEME Suite web site
(using the --enable-web
option in the ./configure command),
you may optionally define a collection of sequence databases.
This will define what sequence databases the associated MEME Suite web services
will maintain and allow users to search. **If you do not do this,
you will not be able to use the sequence search tools on your website.**

1. Firstly, you must create the list of sequence databases on the **web site**
   using the commands:

   $ cd etc
     
   $ get\_db\_csv -db GENBANK -class Bacteria > db\_genbank\_bacteria.csv
     
   $ get\_db\_csv -db GENBANK -class Fungi > db\_genbank\_fungi.csv
     
   $ get\_db\_csv -db ENSEMBL > db\_ensembl.csv
     
   $ get\_db\_csv -db ENSEMBL\_ABINITIO > db\_ensembl\_abinitio.csv
     
   $ get\_db\_csv -db UPSTREAM > db\_upstream.csv
     
   $ cat db\_general.csv db\_other\_genomes.csv db\_ensembl.csv db\_ensembl\_abinitio.csv db\_genbank\_fungi.csv db\_genbank\_bacteria.csv db\_upstream.csv > fasta\_db.csv
     
   $ make install
     
     
   **You should rerun the above commands periodically in order to have the latest
   list of available sequence databases.**
     
     
   You can also edit the
   fasta\_db.csv
   file to add or remove sequence databases. (Place a "#" in the first position
   of a line to remove a sequence database.)
   Alternatively, you can create your own
    fasta\_db.csv  by hand using
   a spreadsheet program. The meaning of each column is given
   in the first line of each of the db\_\*.csv
   files included in the distribution.
2. Next you must
   update (download) the sequence databases on the MEME Suite **web services**
   host (where you installed the web services using
   the --enable-webservice
   option in the ./configure command).

   The possible commands to update the sequence databases are:
     
   $ update\_db > dblog.txt
     
   $ update\_db -upstream > dblog.txt
     
   $ update\_db -upstream -noclobber > dblog.txt
     
   These commands update, respectively,
   1) all sequence databases that do not end with the suffix '\_upstream';
   or 2) all sequence databases including '\_upstream' ones;
   or 3) all sequence databases plus '\_upstream' ones that have not yet been downloaded.
   Note that to use '-upstream' you must create the link
   'your-install-path/lib/perl/RSATWS'. This link should point to
   the RSATWS perl module library
   available from 
   http://rsat.scmbb.ulb.ac.be/rsat/web\_services/RSATWS.tar.gz.

   The resulting files in the db/ directory may be many gigabytes,
   depending on the contents of the
   fasta\_db.csv
   file **on the web site host** (not the one on the **web services host**).
   It can take time to download all the sequence databases, and the connections to some
   sites from which files are downloaded may be not always available.
   Check the output of the script (dblog.txt) for error messages.
   If there are errors,
   the command to install the sequence databases will need to be repeated.
   The script will skip downloading 'normal' sequence databases that are current with the
   database source, and 'upstream' sequence databases that have already been
   downloaded if '-upstream -noclobber' is specified.

   In order to keep the sequence databases current, you can place one of
   the above 'update\_db' commands
   in a chron job that runs at regular intervals.

[ Top ]

---

## Installing GOMO databases

GOMO uses additional databases which need to be downloaded and installed.

1. Download databases for GOMO from here.
2. Extract the databases from the gziped tar:
3. Copy the file
   gomo\_databases/gomo\_db.csv
   to the source
   etc
   directory and proceed though
   the normal installation steps until you have run:
4. Move the extracted databases folder (or create a soft link) into the installed
   db/
   directory ensuring that the folder (or soft link) in the
   db/
   directory is named
   gomo\_databases.

[ Top ]

---

## Installing motif databases

TOMTOM uses additional databases which need to be downloaded and installed.

1. Download the motif databases from here.
2. Extract the databases from the gziped tar:
3. Copy the file
   motif\_databases/motif\_db.csv
   to the source
   etc
   directory and proceed though
   the normal installation steps until you have run:
4. Move the extracted databases folder (or create a soft link) into the installed
   db/
   directory ensuring that the folder (or soft link) in the
   db/
   directory is named
   motif\_databases.

**Note:**Due to copyright restrictions, some motif databases
may be absent from the file you downloaded in step 1. You may
"comment out" the line in the
gomo\_databases/motif\_db.csv file
for a missing database by adding a "#" in front of the line
for that databases using a text editor. This will inactivate
that motif database.
Alternatively, if you own a license for a missing database,
you can convert the database to MEME format using a command-line script provided
with the MEME Suite. These scripts are documented under
"motif database conversion utilities", and have
names that begin with the motif database name and end with
2meme.

[ Top ]

---

## Known issues


[ Top ]

---

## Reporting problems

Make sure first that you really have a problem:

- **1. Check your environment**
- **Look for the answers.**
- **Are you asking in the right place?**
- **Verify what you did.**
- **Report the problem.**

[ Top ]
